# Evaluation of clinical effect of transabdominal sonography and transvaginal sonography in early diagnosis of ectopic gestation

**DOI:** 10.12669/pjms.331.11444

**Published:** 2017

**Authors:** Yinghui Li, Tao Feng, Jiandong Sun

**Affiliations:** 1 Yinghui Li, Dept. of Health Examination, Binzhou People’s Hospital, Shandong 256610, China; 2 Tao Feng, Dept. of Special Inspection, Binzhou People’s Hospital, Shandong 256610, China; 3 Jiandong Sun, Dept. of Special Inspection, Binzhou People’s Hospital, Shandong 256610, China

**Keywords:** Ectopic gestation, Transabdominal sonography, Transvaginal sonography

## Abstract

**Objective:**

To analyze the application of transabdominal sonography and transvaginal sonography in early diagnosis of pregnancy and to provide a reference for the selection of diagnosis methods in clinic.

**Methods:**

One hundred and eighty patients who were admitted into the hospital from February 2013 to February 2014, received clinical diagnosis and were confirmed as ectopic gestation by surgery were selected as research objects. They were examined by transabdominal sonography, transvaginal sonography, and transabdominal sonography in combination with transvaginal sonography respectively. Besides, the ultrasonic performance was observed and compared with surgical pathological results to analyze and compare diagnostic accordance rate.

**Results:**

The positive rate of transabdominal sonography in combination with transvaginal sonography was much higher than that of transabdominal sonography and transvaginal sonography and the differences had statistical significance (P<0.05). The detection rate of different positive characteristics using the combined examination was much higher than that of transabdominal sonography and transvaginal sonography, and the differences had statistical significance (P<0.05). The detection rates of different types of ectopic gestation and different sizes of mass using transabdominal sonography in combination of transvaginal sonography were higher than that of transabdominal sonography and transvaginal sonography (P<0.05).

**Conclusion:**

Transabdominal sonography in combination with transvaginal sonography can complement information, improve detection rate, and reduce or avoid misdiagnosis and missed diagnosis, which provides a scientific basis for the formulation of clinical treatment scheme.

## INTRODUCTION

Early pregnancy is the most important stage for the development of fetus. Ultrasonic examination indications for early pregnancy include the existence of uterine pregnancy, the number of gestation sac, and the existence of ectopic gestation.^1^ Ectopic gestation is a commonly seen acute abdominal disease and also a cause for the death of pregnant women. Gestation refers to the process of implantation and development of fertilized ovum. If fertilized ovum implants in the site beyond uterus, then it is called ectopic gestation.^2^,^3^ Currently, the incidence of ectopic gestation tends to be higher year by year,^4^ and the disease is not specific in the early stage. But massive haemorrhage may be induced if gestational sac breaks; and adverse symptoms such as acute abdominal disease and shock can also happen in severe case, which can severely threaten the life safety of patients.^5^ Therefore, early diagnosis is of great significance.

Currently, ectopic gestation is mainly diagnosed by transvaginal sonography and transabdominal sonography; the two kinds of examination have their own features; most patients with ectopic gestation can be diagnosed before rupture, but misdiagnosis or missed diagnosis is still possible.^6^ Transabdominal sonography, a traditional examination method for ectopic gestation, is featured by wide scanned area. Transabdominal sonography can rapidly and accurately detect ascites, but the examination result is of high risks to be affected by obesity and insufficient filling of bladder, which can significantly lower the diagnostic accuracy of ectopic gestation.^7^,^8^ Transvaginal sonography, a kind of intracavity examination, can clearly display the location and condition of lesions as well as the fine structure and characteristics of uterine cavity and adnexa area by diving a high-frequency probe into vagina and moreover it is free from the influence of obesity, pneumatosis and insufficient filling of bladder. It can display both the amount and property of pelvic fluid and distinguish gestational sac and adnexal mass. It demonstrates more obvious advantages especially when the diameter of a lesion is smaller than 25 mm.^9^,^10^

To compare the clinical effects of transvaginal sonography, transabdominal sonography and transvaginal sonography in combination with transabdominal sonography, one hundred and eighty patients with ectopic gestation who were admitted into the Binzhou People’s Hospital were selected and grouped for study.

## METHODS

### General data

One hundred and eighty patients who received clinical diagnosis in the hospital from February 2013 to February 2014 and were confirmed as ectopic gestation by surgery were selected as research subjects. The onset-to-door time of the patients ranged from one hour to seven days They aged from 20 to 36 year-old (average 27.2±2.2 year-old). The duration of menopause of the patients was 32 ~ 49 d (average 40.6±9.7 d). Of 180 women, 124 women were primiparas and 56 women were multiparas. The major clinical symptoms included menopause, irregular vaginal bleeding, stomachache, abdominal mass and syncope. All patients signed informed consent before study.

### Methods

GE LOGIQ S7 color Doppler ultrasonic diagnostic apparatus was used. The frequency of transvaginal probe was 6.0 MHz and the frequency of abdominal probe was 3.5 MHz. One hundred and eighty patients all underwent transvaginal sonography and transabdominal sonography.

As to transabdominal sonography, after the bladder was filled moderately, conventional examination was performed using abdominal probe with a frequency of 3.5 MHz to detect the endometrial morphology and the blood flow, the size and location of uterus, the presence of fluid in pelvic cavity, hepatorenal recess, bilateral fossa iliaca and splenorenal recess and around liver, and the presence of enclosed mass in Douglas pouch and its bilateral fossa iliaca. If there was fluid, then the involved scope and sound transmission condition were observed. If there was enclosed masses, then the correlation of the morphology, size, boundary and internal echo of the enclosed mass with surrounding organs as well as the blood flow of bilateral adnexa area and trophoblast around enclosed masses were observed.

As to transvaginal sonography, the bladder was emptied at first and then patients were examined in a lithotomy position. Before examination, the top of the probe was smeared with coupling agents and then covered by a sterile condom. The probe whose frequency was set as 6.0 MHz was slowly sent into the vagina. Multidirectional examination was performed by inclining, pushing, pulling and rotating the probe. Adnexal mass, embryo, pelvic fluid, primitive cardiac tube pulse and intrauterine phantom pregnancy bursa were observed. For patients who experienced cesarean delivery, the site with anterior wall scar was paid more attentions, in order to avoid the missed diagnosis of cesarean scar pregnancy.

### Criteria of ultrasonic diagnosis

Ultrasonic diagnostic result could be determined as positive when any of the following conditions was observed: there was no gestation sac in utero, but gestation sac echo which manifested as strong echo mass surrounded by an echo-free sac was detected beyond utero; adnexa area and the envelop of gestational sac were complete, with embryo and original cardiovascular pulsation inside; adnexa area on one side was detected with irregular echoes with unclear boundaries and uneven echo masses.

### Statistical analysis

SPSS version 20.0 software was used for data processing. Categorical data were expressed as rate (%) and tested by Chi-square test. Difference was considered to be statistically significant if P<0.05.

## RESULTS

### Image analysis

**Fig. 1:**
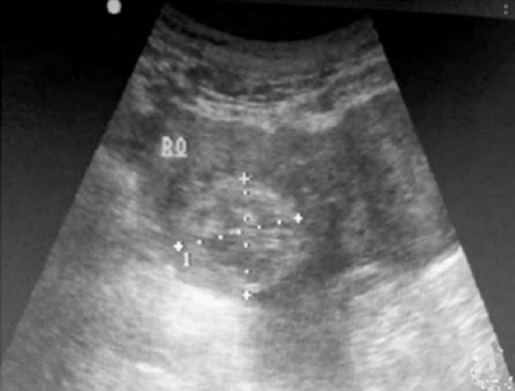
Results of transabdominal sonography: ectopic gestation mass with enhanced echo in adnexa area.

**Fig. 2:**
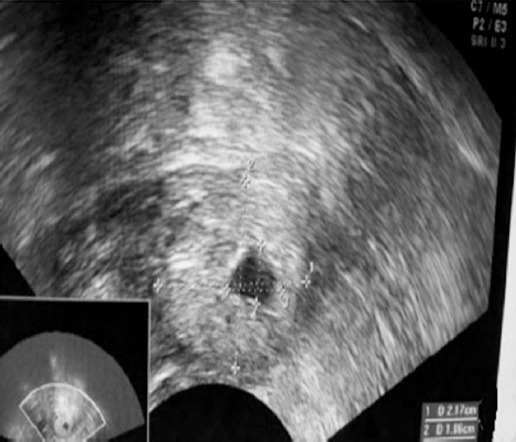
Results of transvaginal sonography: ectopic gestation mass in adnexa area; no obvious gestation sac echo; fuzzy boundary with ovary.

Transabdominal sonography could confirm the existence of gestational sac, but could not accurately diagnose the location and size of gestational sac and the existence of embryo; besides, the diagnosis of the existence of yolk sac, original heart beat, endometrial thickness, the existence of pelvic fluid, blood supply of mass and ascites was not clear (Fig.1). In contrast, transvaginal sonography could accurately diagnose the existence of gestational sac and yolk membrane as well as the location and size of gestational sac, clearly display embryo, sense original heart beat of fetus and detect out endometrial thickness and the depth of pelvic fluid (Fig.2).

### Comparison of diagnosis accordance rate

Ultrasonic testing results of one hundred and eighty patients with ectopic gestation were as follows. One hundred and sixty-two patients were confirmed with ectopic gestation by transvaginal sonography, and the positive correct diagnosis rate was 90.0%; one hundred and forty-three patients were confirmed with ectopic gestation by transabdominal sonography, and the positive confirmed diagnosis rate was 79.4%; one hundred and seventy-four patients were confirmed with ectopic gestation by transvaginal sonography in combination with transabdominal sonography, and the positive confirmed diagnosis rate was 96.7%. The detection rate of the combined detection was higher than that of transabdominal sonography and transvaginal sonography, and the differences had statistical significance (P<0.05; Table-I).

**Table-I:** Comparison of positive confirmed diagnosis rates of ectopic gestation using transabdominal sonography, transvaginal sonography and the combined examination.

Group	N	No. of confirmed cases	Positive correct diagnosis rate (%)
Transabdominal sonography	180	143	79.4
Transvaginal sonography	180	162	90.0
Combined examination	180	174	96.7

### Comparison of positive detection rates of imaging characteristics

**Fig. 3:**
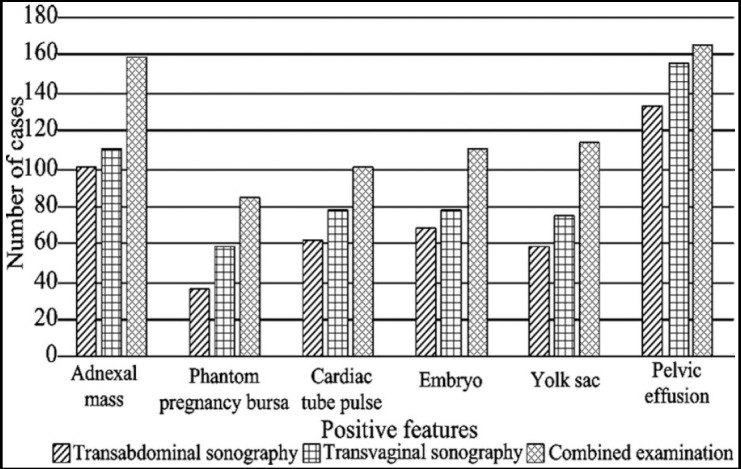
Comparison of detection rates of positive characteristics using transabdominal sonography, transvaginal sonography and the combined examination.

The detection rates of different imaging characteristics using the combined detection were much higher than those of transabdominal sonography and transvaginal sonography (Fig.3).

### Comparison of detection rates of different types of ectopic gestation

The detection rates of ruptured-type, abortion-type and non-ruptured type ectopic gestation using transabdominal sonography in combination with transvaginal sonography and transvaginal sonography were much higher than using transabdominal sonography (P<0.05). The detection rates of various types of ectopic gestation using the combined detection were the highest (Table-II).

**Table-II:** Comparison of detection rates of different types of ectopic gestation using transabdominal sonography, transvaginal sonography and the combined examination.

Type	Pathological diagnosis result	Transabdominal sonography	Transvaginal sonography	Combined examination
Ruptured type	108	94(87.0%)	101(93.5%)	104(96.3%)
Abortion type	38	31(81.6%)	33(86.8%)	37(97.4%)
Non-ruptured type	34	18(52.9%)	28(82.3%)	33(97.1%)

Total	180	143(79.4%)	162(90.0%)	174(96.7%)

### Comparison of detection rates of different sizes of mass

In the detection of different sizes of mass, the detection rate of mass using transabdominal sonography in combination with transvaginal sonography was much higher than that using transabdominal sonography and transvaginal sonography (P<0.05; Table-III).

**Table-III:** Comparison of detection rate of different sizes of mass using transabdominal sonography, transvaginal sonography and the combined examination.

Diameter	Pathological detection results	Transabdominal sonography	Transvaginal sonography	Combined examination
<3cm	65	44(67.7%)	55(84.6%)	61(93.8%)
3cm~5cm	82	69(84.1%)	75(91.5%)	80(97.6%)
>5cm	33	30(90.9%)	32(97.0%)	33(100%)
Total	180	143(79.4%)	162(90.0%)	174(96.7%)

## DISCUSSION

The incidence of ectopic gestation has become increasingly higher year by year with the heavier working and living pressure and the higher incidence of gynecologic inflammation and induced abortion; in addition, there are more and younger patients.^11^,^12^ Fallopian tubes is a site that ectopic gestation frequently happens, with an incidence of 95%. Massive haemorrhage may occur once gestational sacs in fallopian tube break, which may threaten the lives of patients and induce secondary infertility if no timely rescue and treatment are adopted.^13^,^14^ Thus, the early diagnosis of ectopic gestation is the key of treatment. Currently, ultrasonic examination as an invasive examination has become the main means for the diagnosis of ectopic gestation, among which, transvaginal sonography and transvaginal sonography featured by simple operation, high repeatability, and high accuracy can provide patients with effective and important information about treatment and prognosis.^15^,^16^

In this study, transabdominal sonography and transvaginal sonography were combined for diagnosis. The results demonstrated that, the detection rate of the combined examination was 96.7%, much higher than that of transabdominal sonography and transvaginal sonography, and the differences had statistical significance (P<0.05); the detection rates of various positive characteristics using the combined examination was also much higher than those of transabdominal sonography and transvaginal sonography, and the difference was statistically significant (P<0.05). For those who are suspected with ectopic gestation but are not observed obvious abnormality by transabdominal sonography, transvaginal sonography should be used as well. When the location of mass is found to be high by transvaginal sonography or the uterus is in a high position or has a large volume, transabdominal sonography is suggested to be used to confirm ectopic gestation.

In addition, it was also found that, the detection rate of ruptured-type ectopic gestation, abortion-type ectopic gestation and non-ruptured type ectopic gestation using transabdominal sonography was 87.0%, 81.6% and 52.9% respectively; the detection rate of ruptured-type ectopic gestation, abortion-type ectopic gestation and non-ruptured type ectopic gestation using transvaginal sonography was 93.5%, 86.6% and 82.3% respectively; missed diagnosis occurred in both conditions. But the detection rate of ruptured-type ectopic gestation, abortion-type ectopic gestation and non-ruptured type ectopic gestation using the combined examination was the highest (96.3%, 97.4% and 97.1%); the difference had statistical significance (P<0.05). In this study, the diagnosis of 26 cases was missed when transabdominal sonography was used, and most of them were non-ruptured type. That was because the lesions of patients who once experienced pelvic inflammation or cesarean delivery were difficult to be displayed due to pelvic adhesion, severe aerenterectasia and low-degree filling or excessive filling of the bladder.^17^ But transvaginal sonography is free from the interference of gas. The diagnosis of 13 cases was missed due to the high location of lesions when transvaginal sonography was used. It has been found that, transvaginal sonography is more effective in displaying far-field images because of its low frequency and good penetrability of probe,^18^ while transabdominal sonography has more advantages in diagnosing ectopic gestation with highly distributed, ruptured or large masses. Moreover, the comparison of the detection results of different sizes of mass suggested that, the detection rate of mass using the combined examination was the highest, indicating the combined detection could improve the diagnosis accuracy and reduce misdiagnosis and missed diagnosis. This work provides a more scientific basis for the formulation of clinical treatment scheme.

## CONCLUSION

Transabdominal sonography in combination with transvaginal sonography can provide an effective basis for the diagnosis of etopotic gestation. Early diagnosis and treatment are what doctors expect. The combination of transabdominal sonography and transvaginal sonography complements the defects of two methods and thus improves the accuracy of diagnosis. The combined examination method plays a guidance role in the early diagnosis of etopotic gestation and has an important clinical significance.
